# Influence of Physical Activity and Cup Orientation on Metal Ion Release and Oxidative Stress in Metal-on-Metal and Ceramic-on-Metal Total Hip Arthroplasty

**DOI:** 10.3390/jcm13020527

**Published:** 2024-01-17

**Authors:** Jorge Gómez-Álvarez, Ignacio Miranda, Alejandro Álvarez-Llanas, Juan F. Lisón, Francisco Bosch-Morell, Julio Doménech

**Affiliations:** 1 Department of Orthopedic Surgery, Clínica Universidad de Navarra, 31008 Pamplona, Spain; jgomeza@unav.edu; 2 Department of Orthopedic Surgery, Hospital Arnau de Vilanova, 46015 Valencia, Spain; 3 Faculty of Health Sciences, Universidad Católica de Valencia, 46001 Valencia, Spain; 4 Department of Biomedical Sciences, Faculty of Health Sciences, University CEU Cardenal Herrera, 46115 Alfara del Patriarca, Spain; juanfran@uchceu.es (J.F.L.); fbosch@uchceu.es (F.B.-M.); 5 CIBER de Fisiopatología de la Obesidad y Nutrición, Instituto de Salud Carlos III, 28029 Madrid, Spain

**Keywords:** metal on metal, total hip arthroplasty, oxidative stress, metallosis, ceramic on metal

## Abstract

Background: Metal-on-metal (M-M) total hip arthroplasty (THA) has shown adverse reactions to metal debris, abnormal soft-tissue reactions, and high blood metal ion levels. This study aims to: (1) assess whether the toxicity of high levels of ions is related to altered oxidative stress and (2) evaluate tribological factors related to increased blood levels of chromium (Cr) and cobalt (Co) ions. Methods: A cross-sectional analytical descriptive study was conducted on 75 patients. A total of 25 underwent M-M THA, 25 ceramic-on-metal (C-M) THA, and 25 were on the THA waiting list. Ion metallic levels in blood, oxidative stress, physical activity, and implant position were compared. Results: In the M-M group, Co and Cr levels were significantly higher than those found in the C-M group and the control group (*p* < 0.01). We found no differences in terms of oxidative stress between the groups. Also, we did not find a correlation between metal blood levels and oxidative stress indicators, the physical activity of the patients or the position of the implants between groups. Conclusions: The use of M-M bearing surfaces in THA raises the levels of metals in the blood without modifying oxidative stress regardless of the physical activity levels of the patients. Therefore, although patients with M-M bearings require close monitoring, it does not seem necessary to recommend the restriction of physical activity in patients with M-M or C-M arthroplasties.

## 1. Introduction

In the 1950s, McKee and Watson-Farrar introduced the first metal-on-metal (M-M) total hip arthroplasty (THA) [1], but its use did not spread because of the expansion of arthroplasty with metal-on-polyethylene bearing surfaces. However, in the 2000s, the second generation of M-M THA gained popularity as an alternative for younger patients. It was estimated that 35% of all THAs utilized M-M bearing surfaces in the United States in the years after its invention [2]. Follow-up studies of large-head M-M bearings have shown adverse reactions to metal debris (ARMD) [3,4,5,6], reports of abnormal soft-tissue reactions to M-M implants, and elevated blood metal ion levels [7,8,9]. However, some series report good results despite showing elevated Cr and Co levels in patients with M-M THA [10,11] or M-M hinge mechanism in total knee arthroplasty [12]. In vitro studies with ceramic-on-metal (C-M) bearings have shown reduced friction and wear compared to M-M bearings but with a large variation in metal ion levels [13] However, the 2023 UK National Joint Registry [14] established that of the 1,448,541 total hip replacements in the last 20 years (2003–2022) in England (including the Isle of Man and Guernsey), Wales and Northern Ireland, 74,006 received M-M bearings and 2143 received C-M bearings. Furthermore, it was estimated that over one million patients worldwide have received an M-M hip implant [15]. Extensive research has linked increased metal ions to local soft tissue reactions described as ARMD (pseudotumor, metallosis and cell death, and peri-prosthetic tissue necrosis) [16,17,18]. In cancer patients, Chromium (Cr) has been observed to induce oxidative stress, cause alterations in DNA methylation, and potentially result in changes in gene expression and cellular physiology [19]. Additionally, although rare, cases of neurological, musculoskeletal or cardiovascular toxicity associated with high levels of cobalt (Co) have been reported in patients with THA M-M bearing surfaces [20,21,22,23]. However, the mechanism by which toxicity is mediated is still not well understood, nor is the reason why some patients have higher levels than others regardless of renal function. Chromium (Cr) and Co—as redox-active metals—have been shown to be a source of reactive oxygen species (ROS) and various in vitro and in vivo evidence suggests that the toxicity is mediated by the production of ROS and, consequently, oxidative stress [23,24,25,26]. ROS are highly reactive molecules containing oxygen, such as free radicals, generated during cellular processes like metabolism. Oxidative stress occurs when there is an imbalance between the production of ROS and the cell’s ability to detoxify them, leading to cellular damage. Prolonged oxidative stress is implicated in various diseases, aging, and the deterioration of cell health, as it can harm vital cellular components and disrupt normal physiological functions [27]. Lipid peroxidation, a consequence of free radical damage, directly harms membranes and generates aldehydes like malondialdehyde (MDA), the most abundant aldehyde from lipid peroxidation [26]. Oxidative stress reduces the total antioxidant capacity (TAC), and the resulting cascade of free radicals causes lipid peroxidation, DNA damage, cell death, and neurological problems [28].

A possible relationship has been suggested between the position and size of the THA components and the level of physical activity of the patients with a higher release of ions and higher levels of Co and Cr in the blood [29,30,31,32,33,34]. In any case, these studies measure the level of activity by means of activity questionnaires that could offer unreliable results, so the use of other measuring elements, such as the use of accelerometers, would be a good option to obtain more accurate measurements [35].

The objectives of the present study were: (1) to determine whether the C-M pair is susceptible to generating fewer ionic particles than the M-M pair; (2) evaluate whether the toxicity of high levels of ions is related to an alteration of oxidative stress; and (3) evaluate the influence of physical activity and tribological factors on Cr and Co blood-level elevation. Our initial hypothesis was that high concentrations of serum Cr-Co in patients with THA alter cellular oxidative stress, analogous to that observed in subjects exposed to heavy metals in industrial environments. In addition, we also hypothesized that higher levels of Cr-Co in the blood could be related to the position and size of the THA components and the physical activity level of the patient.

## 2. Materials and Methods

A cross-sectional observational analytical descriptive study was carried out. A total of 3 groups of 25 subjects each were formed: the first was subjects who underwent M-M hip arthroplasty, the second was subjects who underwent C-M hip arthroplasty, and the third was the control patient group. Patients undergoing hip replacement were randomly recruited from a cohort of 275 patients operated on by the same surgeon between 2006 and 2010 at the Arnau de Vilanova Hospital in Valencia. The patients in the control group were selected from the surgical waiting list for THA patients diagnosed with osteoarthritis.

Inclusion criteria were adult men or women, aged 40 to 80 years, with coxarthrosis (Tönnis grade 2–3) [36]. The two groups with THA had a minimum time from surgery of 3 years. Exclusion criteria were revision surgery, conditions that can elevate Cr-Co blood levels (osteosynthesis implants, knee or shoulder prostheses, dental implants, work in metal factories, chronic treatment with B12 vitamin or nephropathy), conditions that can elevate oxidative stress (infection or cancer), and patients with missing data.

In all M-M patients, the cup was ASR with a modular ASR-XL head (DePuy Synthes, Warsaw, IN, USA). The C-M patients had undergone implantation of a PINNACLE cup with a 36-mm cobalt-chrome insert and a 36-mm BIOLOX Delta ceramic head (DePuy Synthes, Warsaw, IN, USA). In both groups the femoral stem was Proxima (DePuy Synthes, Warsaw, IN, USA). To verify the implant position, the acetabular component angle was measured. Centricity Universal Viewer Version 6.0 (Barrington, IL, USA) was used for angle measurements. Cup inclination measurements were obtained with respect to the bi-ischial line in the posteroanterior radiograph, and the acetabular cup and head size were registered. Patients were asked to rate their pain over the past month on a 100-mm visual analog scale, from 0 (no pain) to 100 (worst pain imaginable) [37]. To assess postoperative function, the Harris Hip Score was used in all patients. This questionnaire has been validated in Spanish to measure disability after THA, with 100 being the best result [38].

### 2.1. Ions Level Determination

To measure blood levels of Co-Cr, a high-resolution inductively coupled plasma mass spectrophotometry (Agilent Technologies, Tokyo, Japan) was used at Cerba International Laboratories Ltd. (Sabadell, Barcelona, Spain). Three ml of blood was extracted and stored in two tubes: one with lithium heparin that was refrigerated for Co levels and another without metals for Cr levels. The minimum detection values were 28.8 nmol/L for Cr and 4.24 nmol/L for Co. The results were expressed in International System units—nmol/L (amount of substance); however, the specific literature [39] usually expresses them using the conventional system of measurements in µg/L (weight of the substance). To express our results, we used the conventional system while applying its specific conversion factors [40]: Cr 1 µg/L = 19.232 nmol/L and Co 1 µg/L = 16.968 nmol/L.

### 2.2. Oxidative Stress Determination

To determine the oxidative stress and cellular damage, 10 mL of blood was extracted and immediately centrifuged, dividing the serum into 5 aliquots. The resulting samples were frozen at 80 °C. MDA and TAC were measured to reflect oxidative stress and cellular damage. For the determination of MDA, Richard’s modified method with the HPLC technique (high-resolution liquid chromatography) (HPLC System, Waters, Milford, MA, USA) was used. The TAC of each patient’s serum was determined using the ELISA kit-Antioxidant Assay Kit (Cayman Chemical, Ann Arbor, MI, USA).

### 2.3. Physical Activity Measurement

The physical activity level of the patients was assessed in two ways: subjectively using activity questionnaires and objectively using an accelerometer. The University of California Los Angeles activity scale (UCLA test) describes 10 activity patterns, and the patient chooses the one that best suits their profile. Level 10 corresponds to “regularly performing impact sports such as jogging, tennis, or skiing” and level 1 to “complete inactivity dependent on others without leaving home”. This questionnaire has excellent psychometric properties and is widely used and recommended [41]. The International Physical Activity Questionnaire (IPAQ) is an instrument to obtain internationally comparable data on health-related physical activity [35,42]. In the present study, the Spanish version [43] of the short version, which consists of 7 questions, was applied. The questionnaire asks the patient about the frequency and duration in the last week of vigorous, moderate, or walking physical activity. The instrument allows users to calculate the physical activity index, whose value corresponds to the product of intensity, frequency, and duration of the activity. The unit created to express the results of this product is METs-min-week which classifies subjects into 3 categories: high (>3000), moderate (600–3000), and low (<600).

Additionally, the patients wore an accelerometer (Actigraph TM GT1M activity monitor—ActiGraph, Pensacola, FL, USA) 24 h a day, and data were collected every minute for a period of one week [35]. The accelerometer was attached to a flexible belt around the user’s waist. Patients were instructed to always wear the accelerometer, except for when sleeping or bathing. The correct functioning of the accelerometers was verified by plotting the data obtained from each patient on a graph to confirm the correct appearance of a 7-day sleep–wake pattern. For the comparison of results, an average number of counts per minute was found.

### 2.4. Statistical Analysis

The statistical analysis was performed with SPSS software v15.0 (SPSS, Statistical Package for the Social Sciences, Chicago, IL, USA). The descriptive statistical values were calculated and expressed using mean and standard deviation when normality was found and median with ranges when non-normal distributions were found. Dichotomous variables are shown in percentages and raw data. Normality was checked using the Kolmogorov–Smirnov test. To compare the Cr-Co levels among groups, the analysis of variance (ANOVA) test with a post hoc Games–Howell test was used for paired comparisons. To assess the correlation of oxidative stress or the level of physical activity with blood metal ion levels, the Spearman or Pearson correlation was used when appropriate. For the comparison of continuous variables, the Student’s *t* test for independent samples or the Mann–Whitney U test was used depending on the distribution. The significance level was set at *p* < 0.05.

## 3. Results

Demographic and clinical description of the patients from the three groups is shown in Table 1. There were no significant differences in the different parameters analyzed.

M-M were large bearings with a head size ranging from 36 to 53 (average: 41.9, SD: 4.1), and the cup was one piece without a liner ranging from 48 to 60 (average: 54.2, SD: 2.9). All heads in the C-M group had the same size of 36, and in all cases, the cup was modular, with sizes ranging from 50 to 58 (average: 53.7, SD:2.9).

### 3.1. Metal Blood Levels

The average time from intervention to measurements in this study was 7.6 years (range: 4 to 11 years). Patients in the M-M group presented high levels of serum Co that were statistically higher than those found in the C-M group and the control group (Table 2). There were no statistically significant differences when comparing the C-M group with the control group. In the C-M group, 4 (16%) of the 25 patients exceeded 34 nmol/L (2 µg/L), but none of them reached the level of 7 ug/L. In the M-M group, 13 of the 25 patients (52%) exceeded 34 nmol/L (2 µg/L), and 3 were above the safe limit of concern established at 118 nmol/L (7 µg/L) [34]. No patient in the control group exceeded safe levels.

Serum Cr levels increased in the M-M group, and these values were statistically higher than those found in the C-M group and the control group (Table 2). No differences were found in Cr levels between the C-M group and the control group. No patient in the control group exceeded values of 40 nmol/L (2 µg/L), which is considered the lower limit of concern. Three patients in the C-M group slightly exceeded this limit. In the M-M group, three patients exceeded the level of 134 nmol/L (7 µg/L). We found a weak positive correlation between kidney function (blood creatinine and urea levels) and blood Cr levels (Table 3), but no correlation with Co levels.

### 3.2. Oxidative Stress

No significant differences were found in terms of blood oxidative stress markers among the three groups (Table 4). Even when grouping the results of the patients with the two friction pairs (M-M and C-M) compared to the control group, no differences were found. A further correlation analysis was carried out between the oxidative stress markers (TAC and MDA) and the blood ion levels in the THA patients without finding a significant correlation (Table 5).

### 3.3. Implant Positioning and Level of Physical Activity

We found no difference in physical activity scores between M-M and C-M patients. Therefore, data from both groups were pooled to look for a correlation between activity levels and blood metal ions (Table 6). There was no correlation (Spearman’s rho) between the levels of metal ions in the blood and the level of physical activity performed by the patients (measured with an accelerometer, IPAQ questionnaire or UCLA test). There was also no correlation between physical activity and blood metal ion levels when the M-M and C-M groups were analyzed separately (Table 7).

Further analysis was performed to look for an association between metal ions levels and tribological aspects of the implant. All patients in the C-M group had a head size of 36, with an average cup size of 54.3 (range: 50 to 58). Patients with the M-M pair had an average head size of 46.6 (range: 36 to 53) and an average cup size of 54.2 (range: 48–60). The inclination of the cup in the coronal plane of the whole sample was 50.7°, with an SD of 7.3°. No correlation between blood metal ion levels was found with head size, acetabulum size, or positioning angle (Table 7). Regarding the clinical results, no significant differences were found when comparing the functional outcomes measured with the HHS and the VAS pain scale between the groups who received M-M and C-M THA.

## 4. Discussion

Various bearing couples have been used in total hip arthroplasty (THA) depending on the material of the prosthetic head and cup insert, including metal-on-polyethylene (M-P), ceramic-on-polyethylene (C-P), M-M, ceramic-on-ceramic (C-C), and C-M. Friction, lubrication, and wear vary for each bearing type, although lubrication and wear are also influenced by patient characteristics and lifestyle. Hard bearings (M-M and C-C) have lower wear than M-P; the C-P bearing has lower wear than M-P and does not reach the figures for M-M and C-C [44]. There is a concern in the scientific community about patients who were implanted with M-M bearings THA years ago with currently sustained and possibly toxic levels of Cr and Co. Serum Co and Cr values were significantly higher in M-M THA patients than those obtained in the C-M group and control group. A recent systematic review found that patient characteristics, BMI, sex, side, and the time elapsed from the index surgery to the last follow-up did not exert a significant influence on the concentration of Co and Cr in serum in patients who had undergone total hip arthroplasty [45]. There is a wide variety of cut-off values proposed for Co and Cr measurements, ranging from 2 to 7 µg/L [34,46]. A consensus statement established the Co threshold value for clinical concern to be within the range of 2 to 7 µg/L [34,47]. The levels of both Co and Cr ions in our study were in the same range as those reported in other studies [18,31,48]. In our study, we observed a great dispersion in the distribution of levels, being very high in some patients and low in others and without relation to physical activity or orientation of the components. Langton et al. [49], using a sample of 257 patients with M-M THA and with a minimum follow-up of two years, observed that 67 (26.1%) had a serum Co concentration that was greater than 7 μg/L. In our study, the percentage of patients who had a level above 5 µg/L was similar (24%). Additionally, more than half of the patients (52%) with M-M THA were in the range of concern between 34 and 118 nmol/L (2 and 7 µg/L), and 3 (12%) exceeded the upper limit of 118 nmol (7 µg/L). In contrast, metal levels in C-M THA were lower and not significantly different from those in the control group. The C-M bearing surface produces comparatively fewer metal particles, but its appearance on the market has been conditioned by the uncertainty regarding metal waste, relegating it to almost testimonial use [14] despite the good results described by some authors [50,51,52]. No patient in the control group exceeded 2 µg/L both for Co and Cr serum values, which is considered the lower limit of concern.

In our series, a weak correlation was found between Cr levels and creatinine and urea levels and no correlation with Co levels. Cr is excreted by the kidneys and through bile and hair in lower proportion [53]. A recent systematic review found that there is limited and heterogeneous evidence regarding the incidence and associated risk factors of kidney disease in patients with arthroplasty. None of our patients had renal insufficiency. In a large-scale study, no link was found between reduced renal function and higher whole-blood Co and Cr concentrations. Instead, elevated Co and Cr were associated with an improved glomerular filtration rate, indicating better kidney function [54]. Two studies with a 10-year follow-up in patients with M-M bearings found no alteration in markers of kidney function compared to healthy subjects [55,56].

Another potential source of metal ion release is friction in the trunnion. However, we think that the participation of the head–neck interface in the elevation of metal ions in our series represents a minimal or negligible percentage compared to that generated on the head–cup surface. Previous investigations found little evidence of fretting and corrosion in a cohort of well-functioning M-M and C-M [57]. Another possible source of metal ion generation is corrosion. The Cr-Co alloy shows an extremely high degree of corrosion resistance even in environments with elevated pH [58].

It has been suggested that the potential toxicity of increased serum levels of Co and Cr could be related to high levels of oxidative stress. Metal ions have the potential to induce the production of ROS, so they could be the mediating mechanism of toxicity in patients with M-M THA. In a patient undergoing hip replacement revision with elevated blood and urinary concentrations of Cr and Co, an association was reported between metallosis and increased expression of NADPH-Oxidase 4 (NOX4), a well-known compound responsible for ROS generation in muscle tissue [23]. Other studies have suggested a relationship between metallosis and oxidative stress [24,25,59]. According to these reports, our initial hypothesis was that the patients with M-M, with higher Cr and Co levels than the C-M group and the control group, should have greater systemic oxidative stress. The present results show that, in the group of patients who had a THA with M-M bearings, both Cr and Co levels were higher than in the C-M group and the control group. However, no correlation was found between Cr and Co levels and higher oxidative stress indicators. Our results agree with other studies showing the absence of a relationship between Cr and Co blood concentrations and several markers of oxidative stress in patients with M-M THA and resurfacing [48,60,61,62]. Another study comparing M-M hip resurfacing with C-C THA showed increased oxidative stress markers in the resurfacing patients [59]. The discrepancies observed between the aforementioned studies could be related to methodological differences in evaluating oxidative stress. We evaluated oxidative stress by measuring MDA and TAC, as these markers are the most frequent indicators of lipid peroxidation and have been used in other studies [59,63,64,65].

Finally, we have also examined the possible relationship between increased levels of metals and the level of physical activity and component orientation, as suggested by some in vitro [30,66] and clinical [31] studies. Thus, incorrect arrangement of components should elevate metal levels as suggested by in vitro studies [32,33]. However, we did not observe such evidence. The present results show no correlation between the level of physical activity (measured with the UCLA scale, the IPAQ questionnaire, and an accelerometer) or the position of the implant and Cr and Co elevation. Our results agree with other clinical studies that found no relationship between cup inclination and metal ion enhancement in M-M THA [48] or resurfacing [31]. The discrepancy between in vitro and clinical studies may be because in vitro studies expose the components to very high friction cycles in a short time, whereas friction cycles in patients are slower over time. M-M bearings exhibit a biphasic wear pattern with high initial wear in the first few months, followed by a decrease to a stable phase of low ion release that is maintained over time [67]. The patients in our study have been operated on for several years, so they are in a stable phase of ion release. On the other hand, the increase in blood metal ions in THA does not depend solely on M-M friction at the head–cup joint. Another source of mechanical ion release could be corrosion at the head–neck taper junction [68]. In fact, increased Cr and Co levels secondary to corrosion at the head–neck taper junction have also been observed in patients with M-P THA [69]. According to our results, patients with an M-M or C-M prosthesis do not need to limit their level of physical activity.

This study has several limitations. The number of patients (25 per group) is limited and may not have been sufficient to detect differences in oxidative stress among groups. This study is not randomized, so comparing groups could be biased. It is expected that hip arthroplasty patients will have more limited physical activity, so the range of differences in activity from one patient to another may be too small to influence the level of ions released. Patients in the M-M group of this series wore a cup model (ASR) that was withdrawn from the market due to a high retrieval rate. The average follow-up was 7 years, and it is possible that a survival bias occurred and caused us to analyze the cases with better results. However, this study provides valuable information to consider in recommendations to such patients who continue to be followed up in medical consultations.

## 5. Conclusions

In conclusion, our study reveals that the C-M THA configuration produces fewer ionic particles than the M-M counterpart. The use of M-M bearing surfaces in THA raises the levels of metals in the blood without modifying oxidative stress, regardless of the position of the implant (orientation of the cup). No relationship was observed between the level of physical movement and ambulation with increased metal ions in both M-M and C-M groups. Therefore, although patients with such bearings require close monitoring, it does not seem necessary to recommend restricting physical activity in patients with M-M or C-M arthroplasties.

## Figures and Tables

**Table 1:** Demographic and clinical characteristics of the patients.

	M-M	C-M	Control	*p* Value
*n*	25	25	25	n.s.
Women	10 (40%)	10 (40%)	12 (48%)	n.s.
Age (years)	63 (range 46–79)	65 (range 37–82)	62 (range 37–82)	n.s.
Weight (kg)	80.5 ± 16.7	79.6 ± 11.3	79.8 ± 12.6	n.s.
BMI (kg/m^2^)	29 ± 5.1	30.2 ± 4.8	29.4 ± 4.9	n.s.
HHS	86.5 ± 12.7	86.2 ± 9.9	-	n.s.
VAS	2.2 ± 2.6	1.3 ±1.8	-	n.s.

M-M, metal-on-metal; C-M, ceramic-on-metal; BMI, Body Mass Index; HHS, Harris Hip Score; VAS, Visual Analogue scale. Data were expressed as mean ± standard deviation (SD).

**Table 2:** Serum cobalt and chromium levels in patients with metal-on-metal or ceramic-on-metal total hip replacement and in patients from the control group.

Metal Ion	M-M (*n* = 25)	C-M (*n* = 25)	Control (*n =* 25)	*p* Value	
Cobalt (nmol/L)	65.9 ± 58.0	20.1 ± 14.8	5.8 ± 2.7	*p* < 0.001	M-M vs. C-M *p* < 0.001 C-M vs. control *p* = 0.32 M-M vs. control *p* < 0.001
Cobalt (µg/L)	3.8 ± 3.4	1.2 ± 0.8	0.3 ± 0.1		
Chromium (nmol/L)	51.6 ± 39.4	31.1 ± 4.91	29.1 ± 1.8	*p* = 0.001	M-M vs. C-M *p* = 0.04 C-M vs. control *p* = 0.17 M-M vs. control *p* < 0.02
Chromium (µg/L)	2.6 ± 2	1.6 ± 0.2	1.5 ± 0.09		

M-M, metal-on-metal; C-M, ceramic-on-metal. Data are expressed as mean ± standard deviation (SD).

**Table 3:** Correlation between kidney function (creatinine and urea) and Cr and Co levels.

Spearman’s Rho		Creatinine	Urea
Chrome	R	0.367 *	0.334 *
	Sig. (bilateral)	0.017	0.028
Cobalt	R	−0.223	0.179
	Sig. (bilateral)	0.155	0.252

* *p* < 0.05.

**Table 4:** Oxidative stress markers in patients with metal-on-metal or ceramic-on-metal total hip replacement and in patients from the control group.

	Total (M-M and C-M)	M-M	C-M	Control	*p* Value
TAC	4.4 ± 2.3	4.5 ± 2.5	4.25 ± 2.1	5.0 ± 2.3	n.s.
MDA	2.1 ± 1.2	1.90 ± 1.1	2.5 ± 1.2	2.2 ± 1.2	n.s.

M-M, metal-on-metal; C-M, ceramic-on-metal; TAC: total antioxidant capacity; MDA: malondialdehyde. Data are expressed as mean ± standard deviation (SD).

**Table 5:** Correlation between levels of Cr and Co and oxidative stress markers.

Spearman’s Rho		TAC	MDA
Chrome	R	0.204	−0.162
	Sig. (bilateral)	0.13	0.24
Cobalt	R	R	0.073
	Sig. (bilateral)	P	0.59

TAC: total antioxidant capacity; MDA: malondialdehyde.

**Table 6:** Results of the physical activity variables with the three measurement methods used (accelerometer and UCLA and IPAQ questionnaires) in THA patients.

	Mean ± SD	Range
Accelerometer (counts/minute)	156.7 ± 72.3	18–309
UCLA	5.6 ± 1.4	3–10
IPAQ	1.9 ± 0.6	1–3

UCLA, University of California Los Angeles activity scale; IPAQ, International Physical Activity Questionnaire.

**Table 7:** Bilateral correlations between metal ion levels with physical activity measurements according to the three methods used (accelerometer and UCLA and IPAQ questionnaires) and tribological characteristics.

Spearman’s Rho			Accelerometer	UCLA	IPAQ	Head Size	Cup Size	Cup Orientation
M-M	Cr	R	−0.314	−0.227	−0.070	−0.234	−0.311	0.138
		P	0.219	0.380	0.788	0.261	0.131	0.509
	Co	R	−0.216	−0.110	−0.162	0.301	0.037	0.097
		P	0.406	0.675	0.534	0.144	0.861	0.645
C-M	Cr	R	−0.309	0.312	−0.084	.	−0.072	−0.092
		P	0.161	0.169	0.717	.	0.762	0.663
	Co	R	0.001	−0.089	0.243	.	0.242	0.377
		P	0.996	0.701	0.289	.	0.303	0.063

M-M: metal-on-metal; C-M: ceramic-on-metal; Cr, chrome; Co, cobalt; UCLA, University of California Los Angeles activity scale; IPAQ, International Physical Activity Questionnaire.

## Data Availability

The data presented in this study are available upon request from the corresponding author. The data are not publicly available due to privacy.
